# What influences availability of medicines for the community management of childhood illnesses in central Uganda? Implications for scaling up the integrated community case management programme

**DOI:** 10.1186/s12889-015-2525-4

**Published:** 2015-11-25

**Authors:** James Bagonza, Elizeus Rutebemberwa, Tim Eckmanns, Elizabeth Ekirapa-Kiracho

**Affiliations:** Department of Health Policy Planning and Management, Makerere University College of Health Sciences, School of Public Health, Kampala, Uganda; Robert Koch Institute, Berlin, Germany

**Keywords:** Drug, Availability, Childhood Illnesses, Community health workers, Uganda

## Abstract

**Background:**

The integrated Community Case Management (iCCM) of childhood illnesses strategy has been adopted world over to reduce child related ill health and mortality. Community Health workers (CHWs) who implement this strategy need a regular supply of drugs to effectively treat children under 5 years with malaria, pneumonia and diarrhea. In this paper, we report the prevalence and factors influencing availability of medicines for managing malaria, pneumonia and diarrhea in communities in central Uganda.

**Methods:**

A cross sectional study was conducted among 303 CHWs in Wakiso district in central Uganda. Eligible CHWs from two randomly selected Health Sub Districts (HSDs) were interviewed. Questionnaires, check lists, record reviews were used to collect information on CHW background characteristics, CHW’s prescription behaviors, health system support factors and availability of iCCM drugs. Multivariable logistic regression analysis was done to assess factors associated with availability of iCCM drugs.

**Results:**

Out of 300 CHWs, 239 (79.9 %) were females and mean age was 42.1 (standard deviation =11.1 years). The prevalence of iCCM drug availability was 8.3 % and 33 respondents (11 %) had no drugs at all. Factors associated with iCCM drug availability were; being supervised within the last month (adjusted OR = 3.70, 95 % CI 1.22–11.24), appropriate drug prescriptions (adjusted OR = 3.71, 95 % CI 1.38–9.96), regular submission of drug reports (adjusted OR = 4.02, 95 % CI 1.62–10.10) and having a respiratory timer as a diagnostic tool (adjusted OR =3.11, 95 % CI 1.08–9.00).

**Conclusions:**

The low medicine stocks for the community management of childhood illnesses calls for strengthening of CHW supervision, medicine prescription and reporting, and increasing availability of functional diagnostic tools.

## Background

Although progress has been made in reducing under five mortality in Sub Saharan Africa [1, 2], it is still a huge challenge. The rate of decrease of under-five mortality in Uganda from 1990 (160 per1000 live births) to 2006 (137 per 1000 live births) and now 90 per 1000 live births was good, but insufficient to achieve the Millennium Development Goal of 56 per 1000 live births by 2015 [3, 4]. Three quarters of the under five deaths are still due to a handful of causes such as malaria, pneumonia, diarrhea and newborn conditions [5, 6]. Therefore, interventions that promote appropriate treatment of childhood pneumonia, diarrhoea and malaria will lead to major reductions in under five mortality. The integrated Community Case Management (iCCM) of common childhood illnesses (malaria, pneumonia and diarrhea) has been widely adopted as a means to reduce childhood morbidity and mortality by increasing access to essential child health services [7, 8]. Under the iCCM strategy, Community Health Workers (CHWs) are trained to diagnose and treat sick children with malaria, pneumonia and diarrhea in communities. To this end, they are provided with pre-packed medicines which include amoxicillin for treating non severe pneumonia, Artemisinin Combination Therapy (ACT) for uncomplicated malaria, Oral Rehydration Salts (ORS) and zinc for diarrhea. All patients are screened for the three diseases and treatment is administered basing on the results of the examination and diagnostic testing that includes: malaria rapid diagnostic tests, disease history and respiratory rates. This approach can result in a 70 % reduction in mortality from pneumonia in children under age five, and reduce overall malaria mortality by 53 % [9, 10]. Therefore, effective community case management calls for an uninterrupted supply of medicines and equipment necessary to promote early identification and correct treatment of childhood diseases [11–13].

Child health programs often suffer shortages of key products, which suggest that supply chain factors may be adversely affecting outcomes of those programs [14–17]. Poor supply chain management, including limited or none existent stock control and forecasting, means that even though drugs may be available centrally, there can be frequent stock-outs at community level. Indeed supply chain management systems for national health systems coupled with prescriber’s training, experience, use of guidelines and drug utilization rates, often impact on the availability of medicines [18–20].

Since CHWs are the first level of contact between community and the health care system, stock-outs of essential medicines for treating common childhood illnesses may lead to delayed access to care thereby increasing child mortality [21–23]. Although literature on the assessment of drug availability in the formal health sector is widely available [24–26], medicine availability for community case management programmes for childhood malaria, pneumonia and diarrhea has not been fully documented in Uganda. Secondly, many studies addressing barriers of access to essential drugs in developing countries have mainly focused on the supply chain management systems and not on the prescribers’ perspectives and health system support factors such as training and supervision [14, 16, 27]. Given the paucity of data on the availability of drugs among CHWs, the objectives of this study were to document the prevalence and factors influencing availability of iCCM drugs in central Uganda.

## Methods

### Study setting

This study was conducted in Wakiso district. Wakiso lies in the central region of Uganda about 12 km from Kampala, the capital city of Uganda. Wakiso has a population of 1,260,900 [4] and the top five major causes of morbidity in the district are malaria, respiratory tract infections, intestinal worms, skin diseases and diarrhea [4]. Malaria is endemic in the area and the under-five mortality rate for the region is 128/1000 live births [4]. The district is divided into seven Health Sub Districts (HSDs). A HSD is the functional zone of the district health system responsible for the delivery of maternal and child health services, health promotion messages as well as treatment of infectious diseases such as malaria. Within the HSD are peripheral health facilities which supervise CHW’s activities. Community Health workers are provided with pre-packed medicines which include; amoxicillin, Artemisinin-based Combination Therapy (ACT), Oral Rehydration Salts (ORS) and zinc. Amoxicillin is pre packed in two dose/strength formulations: 125 mg (red pack) for children aged 2 months to 12 months and 250 mg (green pack) for children from 12 months to 5 years. Likewise ACTs (Coartem*) is pre packed in two dose formulations: 20/120 mg pack of 6 tablets (yellow pack) for children aged 2 months to 3 years and the 20/120 mg pack of 12 tablets for children between 3 and 5 years. Each CHW takes care of about 25 to 30 households.

### Study design

This was a cross sectional study that employed quantitative methods of data collection and the outcome of interest was drug availability for the iCCM programme. Eligible respondents were CHWs who had been recruited on the iCCM programme for more than six months by the time of the study in Wakiso district and gave informed consent to participate in the study. CHWs who were not available in the district during the study period were excluded.

### Sample size calculation and sampling procedure

The sample size formula by Kish Leslie [28] was used to calculate the required number of CHWs to participate in the study. A sample size of 305 CHWs was calculated based on the following assumptions: a two sided test with a precision of 5 %, and an estimated prevalence of drug availability of 33 % [29]. The seven HSDs in Wakiso were stratified into rural (4 HSDs) and peri-urban (3 HSDs) and two were selected randomly taking one from the rural HSDs and one from the peri-urban HSDs. On average each HSD had about 180 CHWs managing sick children under the iCCM programme. In each Sub County or town council within the selected HSDs, we contacted the health facility managers or health workers responsible for CHWs activities to obtain lists of CHWs responsible for iCCM programme. We located the eligible CHWs in their homes through their supervisors and local council/village leaders.

### Data collection and drug availability

A physical drug count of the four iCCM drugs (ACTs, Amoxicillin, ORS and Zinc) was done on the day of the interview using data extraction sheets and check lists. Pretested semi structured questionnaires were administered to collect information on factors affecting drug availability. This tool captured socio-demographic information: individual, health system support factors that may affect drug availability. A retrospective records review of the iCCM patient registers for the previous 6 months was done to collect data on CHW’s drug prescription behaviors. Specifically we looked at the diagnoses and drugs prescribed by CHWs for each patient seen in the last 6 months to determine whether the prescriptions were appropriate basing on the iCCM treatment guidelines.

A CHW was classified as having drugs when all the four iCCM drugs were present on the day of the survey. Where a CHW only had some or none of the four iCCM drugs, this was classified as no availability of iCCM drugs. In addition, information on individual drug availabilities and total stock-outs of all the four drugs (absence of any drug) was captured.

Data collection tools were pretested and data was collected by five trained research assistants who were supervised by the principal investigator. Data collection tools were translated into the local language (*Luganda*) and back translated by other people without prior knowledge of the instrument.

### Data analysis

Data were double entered in Epi-Info software version 3.3.2 of 2005 and analyzed using STATA 10 (STATA Corp, College Station, TX, USA). Community health worker’s background characteristics and drug availability were summarized descriptively. Drug availability was dichotomized as “YES” if all the four iCCM drugs were available and “No” if only some or none of the drugs were present. Relationships between CHWs background characteristics, health system support factors and drug availability were assessed using odds ratios. A p value of less than 0.05 was taken to represent a statistically significant association. All variables with a p-value of less than 0.2 [30] at univariate analysis as well as variables known to predict medicine availability from literature such as prescriber’s education level, sex, occupation and time since last training on dispensing drugs [31, 32] were used in multivariable regression analysis. However, before conducting multivariable analysis, the existence of multicolinearlity was investigated using the correlation coefficients between each pair of the independent variables. Correlated variables (correlation coefficient value greater than 0.5 with a p-value less or equal to 0.05) were excluded from the logistic regression analysis model. A binary logistic regression analysis with a backward elimination method was done to determine independent factors associated with availability of iCCM drugs. Conclusions were drawn based on the adjusted odds ratios with their corresponding 95 % confidence intervals. Interactions among the independent variables were not statistically significant. In order to determine how well the model fit the data, Pearson chi-squared test (*p* > 0.05) was used to determine the goodness of fit of the model.

### Ethical considerations

Ethical approval to conduct this study was got from Uganda National Council of Science and Technology, Makerere University School of Public Health Higher Degrees Research and Ethics Committee, and Wakiso district health authorities. Written informed consent was obtained from the study participants. Data accruing from the study was kept securely locked at all times and no personal identifiers were used or recorded on any tools used for data collection.

## Results

Of the 303 study participants, only 300 (99.0 %) were included in our analysis. Data from three respondents (1.0 %) were excluded from further analysis on account of having incompletely filled data collection tools. Table 1 shows background characteristics of respondents. Majority 79.7 % of the respondents were females and the mean age was 42.1 years (standard deviation 11.1 years). More than half, 65 % had secondary education as the highest level of education attained while only 14.3 % were in formal employment.

**Table 1:** Background characteristics of respondents

Variable		Respondents (*N* = 300)
		n (%)
Sex	Male	61 (20.3)
	Female	239 (79.7)
Age	20–29 years	28 (9.3)
	30–39 years	85 (28.3)
	40–49 years	100 (33.3)
	50–50 years	77 (25.6)
	>60 years	10 (3.3)
Education	None/primary	68 (22.7)
	Secondary	195 (65.0)
	Tertiary	37 (12.3)
Religion	Catholic	78 (26.0)
	Protestant	121 (40.3)
	Moslem	56 (18.6)
	Others	45 (15.3)
Marital status	Never married	21 (7.0)
	Married	173 (57.7)
	Separated	60 (20.0)
	Divorced	46 (15.5)
Occupation	Unemployed	63 (21.0)
	Formal employment	43 (14.3)
	Self-employment	175 (58.3)
	Others	19 (6.3)

### Availability of iCCM drugs

Table 2 describes availability of iCCM drugs among CHWs. Only 25 respondents (8.3 %) had all the four iCCM medicines while 33 respondents (11 %) had no drugs at all. ACTs/Coartem*-20/120 mg (6 tablet pack) a drug used to treat malaria was the least available (at 3.3 %) of all the iCCM medicines. Amoxicillin was the most prevalent drug at 79 % for the 250 mg dose strength (green pack) and 64.7 % for the 125 mg dose strength (red pack).

**Table 2:** Availability of iCCM drugs among CHWs

Variable	Availability of iCCM drugs (*N* = 300, %)
Number of iCCMDrugs	
All the four iCCM drugs	25 (8.3)
Only three of the iCCM drugs	69 (23.0)
Only two of the iCCM drugs	107 (35.7)
One of the iCCM drugs	66 (22.0)
No iCCM drug at all	33 (11.0)
Type of iCCM drugs	
ACTs 20/120 mg 6 tablets pack (yellow)	10 (3.3)
ACTs 20/120 mg 12 tablets pack (blue)	106 (35.3)
Amoxicillin 125 mg pack (red)	194 (64.7)
Amoxicillin 250 mg pack (green)	237 (79.0)
Zinc 20 mg	79 (26.3)
Oral Rehydration Salts (ORS)	140 (46.7)

### Factors associated with availability of iCCM drugs among CHWs

Table 3 describes associations between health system factors and availability of iCCM drugs at unadjusted analysis. Community health workers who were regularly submitting monthly reports were more likely to have all the four iCCM drugs (OR = 3.48 95 % CI 1.62–9.52) compared to those who were not submitting reports. Community Health workers who had more than 90 % of their prescriptions done appropriately were more likely to have all the iCCM drugs (OR = 3.32 95 % CI 1.33–8.32). Time since last training in dispensing iCCM drugs, supervision from health workers and not having diagnostic equipment (respiratory timer) were not associated with availability of iCCM medicines at crude analysis.

**Table 3:** Association between Health system factors and drug availability among CHWs

Variable	Drug availability (*N* = 300)		OR (95 % CI)	*P* values
	Yes (n, %)	No (n, %)		
Ever treating children above 5 years				
Yes	2 (11.1)	16 (88.9)	1.00	
No	23 (8.2)	259 (91.8)	0.71 (0.15–3.29)	0.660
Ever supplied drugs to care givers who have not brought sick children				
Yes	1 (5.6)	18 (94.4)	1.00	
No	24 (8.5)	257 (91.5)	1.68 (0.15–9.99)	0.829
Training on dispensing drugs				
Within 6 months	1 (4.0)	24 96.0)	1.00	
6 to 12 month	1 (4.4)	22 (95.6)	1.09 (0.06–19.07)	0.953
More 12 months	23 (9.1)	229 (90.9)	2.41 (0.31–18.77)	0.386
Having a respiratory timer				
No	6 (4.7)	121 (95.3)	1.00	
Yes	19 (9.9)	154 (90.1)	2.48 (0.93–7.69)	0.056
Supervision				
No	19 (7.3)	240 (92.7)	1.00	
Yes	6 (14.6)	35 (85.4)	2.16 (0.71–5.88)	0.179
Monthly reporting				
No	9 (4.2)	182 (95.8)	1.00	
Yes	16 (14.7)	93 (85.3)	3.48 (1.62–9.52)	0.011
Appropriateness of prescriptions				
Less than 90 %	7 (4.3)	155 (95.7)	1.00	
More than 90 %	18 (13.0)	120 (86.9)	3.32 (1.33–8.32)	0.006

Table 4 shows results of the multivariable analysis of independent factors associated with iCCM drug availability. When factors were fitted in a logistic regression model for multivariable analysis, 99.5 % (*n* = 298) of respondents were retained in the analysis. Factors that were found to be independently associated with drug availability were: being supervised within the last one month (adjusted OR = 3.70,95 % CI (1.22–11.24), having more than 90 % of prescriptions appropriately done (adjusted OR = 3.71, 95 % CI 1.38–9.96), regular submission of monthly drug reports (adjusted OR = 4.02, 95 % CI, 1.62–10.10), Having a functional respiratory timer as a diagnostic tool (adjusted OR =3.11,95 % CI, 1.08–9.00). In the multivariable model, we adjusted for sex, prescriber’s education level, occupation and time since the last training in dispensing drugs.

**Table 4:** Factors affecting iCCM drug availability among CHWs

Variable	Crude OR (95 % CI)	Adjusted OR (95 % CI)	*P* value
Appropriateness of prescriptions			
Less than 90 %	1.00	1.00	
More than 90 %	3.32 (1.33–8.32)	3.71 (1.38–9.96)	0.009
Having respiratory timers			
No	1.00	1.00	
Yes	2.48 (0.93–7.69)	3.11 (1.08–9.00)	0.036
Monthly reporting			
No	1.00	1.00	
Yes	3.48 (1.62–9.52)	4.02 (1.62–10.10)	0.003
Supervision by Health workers in previous month			
No	1.00	1.00	
Yes	2.16 ( 0.71–5.88)	3.70 (1.22–11.24)	0.021

## Discussion

### Main findings

Overall, this survey showed low availability of drugs necessary to manage common childhood illnesses at community level in central Uganda. Less than 10 % of the CHWs had all the four iCCM drugs for treating malaria, pneumonia and diarrhea at the time of the survey. Availability of iCCM drugs among CHWs was mainly associated with timely submission of monthly drug reports, supervision by health workers, and appropriateness of prescriptions by CHWs, and having diagnostic tools especially respiratory timers.

### Implications for community care

Our results imply that over 90 % of the CHWs could not effectively treat all the childhood conditions targeted under the iCCM policy on the day of the survey. This could negatively impact on the strategy of using CHWs to increase timely treatment of childhood conditions so as to reduce childhood morbidity. In the management of childhood illnesses, both dose strengths of ACTs (Coartem*) are important because children require different dosages on the basis of age and weight, and substitution of doses may compromise the quality of care. The low concurrent availability of the age specific packs of ACTs (Coartem*) may lead to ineffective treatment of malaria and this subsequently promotes irrational drug use by CHWs. Irrational use of medicines can result in incomplete recovery, development of resistance and adverse medicine reactions. For CHWs who provide curative treatment, the continuous supply of drugs is an essential part of their effectiveness and thus replenishment of drug supplies is necessary to maintain the provision curative services in communities [33]. Intermittent drug supplies often lead to great declines in care seeking for CHW services. When CHWs do not have drugs, community members are often aware of the stock-outs and seek care elsewhere [34, 35]. In addition, drug availability is an important component of CHW motivation [21] and indeed, lack of drugs has been shown to decrease their morale and the community’s perception of their effectiveness [29].

Lack of diagnostic equipment may promote presumptive treatment of pneumonia leading to over prescription of amoxicillin. Correct use of respiratory timers not only improves diagnosis and treatment of pneumonia but also leads to rational use of amoxicillin by CHWs [36, 37]. A large number of the children who die from pneumonia do so as a result of inappropriate treatment due to misdiagnosis of symptoms. First level health facility workers and CHWs diagnose pneumonia, primarily through counting respiratory rates of children with cough or difficulties in breathing. However, counting respiratory rates is often challenging and even for highly trained health workers, misclassifications are common. Pneumonia is often misdiagnosed as malaria by CHWs and caregivers in resource poor settings until it develops into a severe stage. Studies show that many developing countries still face significant challenges in the provision of effective health care, diagnosis and treatment of pneumonia [38, 39]. Diagnostic support aids for pneumonia are expected to contribute to improved, more accurate diagnosis and classification of pneumonia.

Community health workers are supposed to ensure that the right information regarding drug consumption and stock at hand is collected and made available to right people who make informed decisions on the resupply of drugs. Regular submission of drug reports enables program coordinators to quantify drug needs based on utilization. This may improve supply reliability and supply chain performance. In addition, it allows identification of drug supply chain bottlenecks that may affect programme implementation. When community level drug needs are not carefully considered and estimated in quantifications and procurements, CHWs are likely to suffer the most from shortages and expiries since they are at the end of the supply chain [17, 40].

Supervisors of CHWs are supposed to provide ongoing support, identify best practices, challenges, provide feedback in a constructive way, in addition to suggesting coping mechanisms. Additionally, they are supposed to review CHW registers and cross check the drug inventory to ensure that drugs are replenished regularly. Through such interactions with CHWs, supervisors are supposed to reinforce CHW’s competencies in case management, drug use and record keeping.

### Implications for research

Although low medicine stock-outs for treating childhood conditions have been reported previously [16, 41], concurrent stock-outs of the age specific packs for ACTs and amoxicillin deserves serious attention as this precludes any form of treatment for malaria and pneumonia. When the age and weight specific drugs are absent, CHWs tend to improvise treatments by dividing larger pack sizes for lower age categories and combining smaller packs for older children [42, 43]. Although this still enables dispensing, it may compromise patient’s adherence and treatment outcomes [44]. Indeed several studies undertaken under the routine conditions of child care have reported high levels of non-adherence [45, 46]. Nevertheless, the possible negative effects of the stock-outs of the age-specific packs on patient’s adherence need further investigation.

Whereas regular submission of drug reports may increase drug availability as demonstrated in this study and other studies in Malawi, Ethiopia and Rwanda [47], the quality of such reports in terms of completeness, accuracy and timeliness should be examined. These attributes of data quality affect the usefulness of such reports for decision making regarding the supply chain for iCCM drugs. One of the challenges that may lead to delays in reporting could be lack of transport to deliver reports to health facilities and communication breakdowns between CHWs and their supervisors. As such, innovative ways to improve communication between CHWs and the formal health system may enhance CHW’s performance. Although supervision is key to CHW’s performance [48], CHW supervision continues to be reported as one of the weakest links in community health programs [49, 50]. Thus it may be worthwhile to investigate key bottlenecks in supervising CHWs so that they are addressed.

### Strengths and limitations

There are some limitations in the data that are worth noting. In this study, drug availability was determined by the presence of iCCM drugs with CHWs on the day of the survey. Day of visit results do not show if iCCM drugs have been in stock continuously, nor whether supply levels are too low or too high to avoid future stock-outs. Missing data or incomplete filling of patient care registers could have affected how the appropriateness of drug prescriptions was assessed. Since this was a cross sectional study, we cannot infer causality. We only we report factors associated with drug availability other than determinants of drug availability. However, this study provides useful information on the availability of iCCM drugs and the associated factors. Understanding and identifying solutions to address drug constraints for the community case management of childhood diseases may yield substantial improvements in programme’s effectiveness, scale up, and impact in reducing child mortality.

## Conclusion

Medicine availability is a challenge for the iCCM program in central Uganda. Under the iCCM program, CHWs are required to submit monthly drugs reports indicating stock levels before receiving new drug stocks. Innovative ways of improving supervision, reporting, prescribing and availability of diagnostic tools may increase availability of iCCM medicines. Improving availability of iCCM drugs among CHWs is vital if iCCM is going to be successful at treating childhood illness, and ultimately, reducing child mortality.
